# Percussive Vibration Therapy as an Adjunct to Conventional Physiotherapy in Chronic Non-Specific Low Back Pain: A Randomized Controlled Trial

**DOI:** 10.3390/jcm15197711

**Published:** 2026-10-06

**Authors:** Elif Esma Bayraktar, Ömer Şevgin, Ainaz Vahedi, Ertuğrul Safran

**Affiliations:** 1 Department of Physiotherapy and Rehabilitation, Faculty of Health Sciences, Acibadem Mehmet Ali Aydinlar University, Kerem Aydinlar Campus, Kayisdagi No. 32, 34752 Istanbul, Türkiye; elif.bayraktar@acibadem.edu.tr; 2 Department of Physiotherapy and Rehabilitation, Faculty of Health Sciences, Üsküdar University, NP Health Campus, Saray, Ahmet Tevfik İleri No. 5, 34774 Istanbul, Türkiye; 3 Department of Physiotherapy and Rehabilitation, Graduate School of Health Sciences, Üsküdar University, Mimar Sinan District, Selman-ı Pak, 34664 Istanbul, Türkiye; ainaz.vahedi@st.uskudar.edu.tr; 4 Department of Physiotherapy and Rehabilitation, Faculty of Health Sciences, Istanbul Gelişim University, Cihangir, Avcılar, 34310 Istanbul, Türkiye; esafran@gelisim.edu.tr

**Keywords:** low back pain, vibration, physical therapy modalities, sleep quality, pain measurement, randomized controlled trial

## Abstract

**Background/Objectives:** Hand-held percussive vibration therapy (PVT) is widely used in musculoskeletal rehabilitation, yet its incremental benefit beyond an active physiotherapy program for chronic non-specific low back pain (CNLBP) is unclear. We investigated whether PVT added to conventional physiotherapy (CPT) improves disability, sleep quality, and pain. **Methods:** This single-center, parallel-group, assessor-blinded randomized trial enrolled 50 adults aged 18–65 years with CNLBP and baseline visual analogue scale (VAS) pain ≥4/10. Participants were allocated equally to PVT + CPT or CPT alone and attended three sessions weekly for eight weeks. The Oswestry Disability Index (ODI) was the primary outcome; Pittsburgh Sleep Quality Index (PSQI) and VAS scores were secondary outcomes. Week-8 scores were analyzed with baseline-adjusted ANCOVA using HC3 robust standard errors. **Results:** Follow-up data were complete for all participants. The adjusted ODI score at week 8 was 2.59 points higher with PVT + CPT than with CPT (95% confidence interval [CI], 0.47 to 4.71; *p* = 0.017), a statistically significant difference in favor of CPT. PSQI scores did not differ between groups (adjusted difference, 0.25; 95% CI, −0.40 to 0.90; *p* = 0.451). VAS scores were lower with PVT + CPT (adjusted difference, −1.15; 95% CI, −1.45 to −0.85; *p* < 0.001). **Conclusions:** PVT did not provide an additional benefit for disability and did not improve sleep quality when added to CPT. The pain difference was a secondary, exploratory finding of uncertain clinical relevance.

## 1. Introduction

Low back pain accounts for more years lived with disability than any other health condition. Global prevalence was estimated at 619 million cases in 2020 and is expected to reach 843 million by 2050 [1]. For most patients, symptoms cannot be attributed confidently to a single nociceptive or structural lesion, leading to classification as non-specific low back pain [2,3]. Persistence beyond three months is associated with restrictions in function and daily activity, reduced quality of life, and frequent sleep disturbance [2,3,4,5].

Current guidelines place education, continued activity, and exercise-based care at the center of chronic non-specific low back pain (CNLBP) management [6]. Exercise can reduce pain and functional limitation relative to no treatment, usual care, or placebo, although additional gains over other conservative approaches are often modest and heterogeneous [7]. This residual burden has encouraged the use of adjunctive modalities alongside active rehabilitation.

Hand-held percussive devices apply repeated mechanical impulses to selected soft-tissue regions and have become common in rehabilitation and sport. Most available evidence, however, comes from short-term investigations in healthy or active populations and focuses on range of motion, flexibility, perceived recovery, or acute muscular responses [8]. Findings from whole-body and other vibration techniques cannot be transferred directly because the mode, dose, and area of exposure differ. Reviews in chronic low back pain suggest possible effects on pain and disability, but estimates vary substantially by vibration method and comparator, and evidence for local vibration remains uncertain [9,10].

Only limited trial evidence is available for hand-held percussive vibration therapy in CNLBP. Two small randomized studies combined percussion with either core-stability exercise or conventional physiotherapy and reported favorable changes in pain or disability [11,12]. Their populations, treatment schedules, comparators, and outcome measures differed, and neither design allowed the device-specific contribution to be distinguished from additional contact and treatment-related expectations. Sleep outcomes have also received little attention when percussion is incorporated into a comprehensive physiotherapy program.

Accordingly, this randomized controlled trial tested the incremental effect of adding hand-held percussive vibration therapy (PVT) to conventional physiotherapy (CPT) over eight weeks in adults with CNLBP. Disability measured with the Oswestry Disability Index was the primary outcome; sleep quality and pain intensity were secondary outcomes.

## 2. Materials and Methods

### 2.1. Study Design, Ethics, and Registration

This single-center, two-arm, parallel-group randomized controlled trial used equal allocation and blinded outcome assessment. Recruitment and treatment took place at a private physiotherapy and rehabilitation clinic in Istanbul, Türkiye, from March 2025 to January 2026. Participants were enrolled on a rolling basis: the first participant was enrolled in March 2025 and completed the eight-week intervention in May 2025, and the last participant was enrolled in November 2025 and completed the intervention and the week-8 assessment in January 2026. Reporting followed CONSORT 2025, the CONSORT guidance for non-pharmacological trials, and the TIDieR checklist [13,14,15].

The study was conducted in accordance with the Declaration of Helsinki [16] and was approved by the Üsküdar University Non-Interventional Clinical Research Ethics Committee (approval No. 61351342/020-879; 28 January 2025). All participants provided written informed consent before enrollment and any study-related assessment or intervention. The trial was prospectively registered at ClinicalTrials.gov before enrollment of the first participant (NCT06840795).

### 2.2. Participants

Adults referred to the outpatient physiotherapy clinic with CNLBP were assessed for eligibility. CNLBP was defined as pain localized between the lower rib margins and the gluteal folds, persisting for at least 12 weeks and not attributable to an identifiable specific spinal pathology [2,3,6].

Participants were eligible if they were aged 18–65 years, had physician-diagnosed CNLBP, reported a baseline pain intensity of at least 4 on a 0–10 visual analogue scale (VAS), were willing to attend the scheduled treatment program, and were able to understand and cooperate with the assessment and intervention procedures.

Exclusion criteria were structural spinal pathology or deformity, recent spinal surgery, chronic bone disease, or a neurological or orthopedic disorder that could interfere with the assessments or treatment program. Participants were also excluded if they used hypnotic medication, had a psychological or cognitive condition that prevented informed participation or completion of the assessments, or were unable to cooperate with the study procedures. The diagnosis of CNLBP and the absence of suspected serious spinal pathology were determined from the medical history, clinical examination, and available medical records.

### 2.3. Randomization, Allocation Concealment, and Blinding

After completion of the baseline assessment, participants were randomly assigned in a 1:1 ratio to either the percussive vibration therapy plus conventional physiotherapy group (PVT + CPT) or the conventional physiotherapy-alone group (CPT). A computer-generated allocation sequence using simple, unblocked, and unstratified randomization was prepared before participant enrollment.

Sequence generation was performed by an independent researcher who was not involved in participant recruitment, baseline assessment, treatment delivery, outcome assessment, or data analysis. Group assignments were placed in sequentially numbered, opaque, sealed envelopes prepared according to the allocation sequence. The envelopes were opened consecutively only after the participant had been enrolled and the baseline assessment had been completed. The original randomization sequence and allocation log were retained to permit verification of sequence generation and implementation.

Participants and treating physiotherapists could not be blinded because of the overt nature of the interventions. Outcome assessments were conducted by a physiotherapist who was blinded to treatment allocation. The data analyst was also blinded to group identity during the principal analyses.

### 2.4. Interventions

Both groups received three individually supervised treatment sessions per week for eight consecutive weeks, corresponding to a planned total of 24 sessions. Treatments were delivered at the study clinic by an experienced physiotherapist. Each conventional physiotherapy session lasted approximately 48–58 min; sessions in the PVT + CPT group were approximately 10 min longer. Attendance at every scheduled session was documented in the clinic’s appointment records, which were used to determine treatment adherence.

Participants allocated to the CPT-alone group received a standardized multimodal program comprising adjunctive electrophysical modalities, supervised exercise, postural education, and advice regarding activities of daily living. The electrophysical component consisted of a hot pack and conventional transcutaneous electrical nerve stimulation (Compex Fit 3.0; DJO France SAS, Mouguerre, France) applied concurrently to the lumbar paravertebral region for 20 min, the stimulation being delivered at 80–100 Hz with a pulse duration of 50–100 μs. Stimulation intensity was adjusted to produce a strong but comfortable sensory perception without visible muscle contraction. Therapeutic ultrasound (Chattanooga Intelect TranSport Ultrasound, model 2782; DJO, LLC, Vista, CA, USA) was then applied bilaterally over the lumbar paravertebral region between L1 and L5 at 1 MHz and 1.0–1.5 W/cm^2^ in continuous mode. Application time ranged from 5 to 10 min according to the treated area, with an approximate total duration of 8 min. The supervised exercise component followed and lasted 20–30 min. Comparable combinations of supervised exercise and adjunctive electrophysical modalities have been evaluated in clinical studies involving individuals with chronic mechanical or non-specific low back pain [17,18]. Electrophysical modalities were treated as adjuncts to, rather than substitutes for, active rehabilitation, consistent with clinical practice recommendations emphasizing exercise and active management for chronic low back pain [6].

The supervised exercise program comprised active mobility exercises for the lumbar spine and lower extremities; stretching of the hamstrings, hip flexors, piriformis, and lumbar extensor muscles; and progressive trunk and hip strengthening. Motor-control and stabilization exercises targeted the transversus abdominis, multifidus, abdominal, paravertebral, and gluteal muscles. The program included pelvic tilting, abdominal drawing-in, bridging, quadruped contralateral upper- and lower-limb elevation, postural training, and functional movement exercises. These components are consistent with exercise approaches commonly used in chronic low back pain rehabilitation, including trunk strengthening, endurance, specific trunk activation, movement-control, and general exercise [6,7].

Stretching exercises were held for 20–30 s and repeated three times. Strengthening and stabilization exercises were generally performed for 10–15 repetitions and two to three sets. Exercise difficulty was progressed according to movement quality, symptom response, and individual tolerance by modifying the number of repetitions, duration of contraction, exercise position, or mechanical demand. Each session concluded with low-intensity stretching and relaxation exercises.

Participants allocated to the PVT plus CPT group received the same conventional physiotherapy program, with the addition of device-assisted percussive vibration therapy using a Theragun PRO Plus device (Therabody, Inc., Los Angeles, CA, USA) fitted with a round attachment head. With the participant positioned prone, PVT was applied bilaterally to the lumbar paravertebral, quadratus lumborum, and gluteal regions using slow, continuous gliding movements and light-to-moderate pressure. Direct application over the spinous processes and other bony prominences was avoided. The device was operated at a single medium setting throughout, corresponding to approximately 30–40 Hz, that is, approximately 1800–2400 percussions per minute. Each session comprised approximately 10 min of application in total, delivered as approximately 5 min per side across the treated regions, and was given before the exercise component. Applied pressure was guided by therapist judgement and was not quantified by any instrumented measure.

Participants in the experimental group consequently received approximately 10 min of additional treatment and therapist contact per session, corresponding to approximately four additional hours of supervised therapist contact across the 24 planned sessions. No sham device or time-matched attention intervention was used.

### 2.5. Outcome Measures

All outcomes were assessed at baseline and at the end of the eight-week intervention by an assessor who was blinded to group allocation. No interim or longer-term follow-up assessments were available in the analysis dataset.

Disability, assessed using the Oswestry Disability Index (ODI), was designated as the primary outcome. The ODI is a ten-item condition-specific questionnaire evaluating limitations in daily functioning associated with low back pain [19]. The validated Turkish version of the instrument was administered [20]. Each item is scored from 0 to 5. The raw 0–50 scores recorded in the dataset were multiplied by two and reported on the conventional 0–100 scale, with higher scores indicating greater disability [19,20]. Scores were derived from the sum of all ten items; no proportional rescaling for unanswered items was applied.

Sleep quality and pain intensity were designated as secondary outcomes. Sleep quality was assessed using the Pittsburgh Sleep Quality Index (PSQI), which evaluates subjective sleep quality over the preceding month across seven components. The global score ranges from 0 to 21, with higher scores indicating poorer sleep quality [21,22].

Pain intensity was assessed using a 10-cm VAS anchored by “no pain” and “worst imaginable pain” [23,24]. Participants marked their current pain intensity on the line, and the distance from the “no pain” anchor was measured and converted to a score ranging from 0 to 10, with higher scores indicating greater pain intensity [23,24].

Two within-person change values were used to provide context for the observed effects: an approximate 1.5-point change on a 0–10 numeric pain rating scale [25] and a 10-point change on the 0–100 ODI scale [26]. The pain value was derived from a numeric rating scale rather than the VAS used in this trial. Neither value was treated as a validated threshold for interpreting the adjusted between-group differences; both were used only as descriptive reference points.

### 2.6. Sample Size

The sample-size calculation was based on the ODI, the primary outcome. A previous randomized study of percussive vibration therapy added to conventional treatment reported a large between-group effect on ODI scores [12]. A more conservative standardized effect size of Cohen’s d = 0.75 was assumed to reduce the risk of overestimating the effect from a small study. With 80% power, a two-sided α level of 0.05, and equal group allocation, approximately 29 participants per group would have been required for an unadjusted comparison. For the planned baseline-adjusted ANCOVA, the required sample was reduced using the 1−ρ2 correction [27]. Assuming a baseline-to-follow-up correlation of 0.50, as supported by empirical evidence from trials using repeated patient-reported outcomes [28], 22 participants per group were required. Allowing for approximately 12% attrition, the target sample was 50 participants, with 25 participants per group.

### 2.7. Statistical Analysis

Statistical analyses were performed using Python version 3.11 with NumPy, SciPy, and statsmodels. The dataset was checked for duplicate records, missing values, out-of-range observations, and internal inconsistencies. Body mass index was calculated from baseline body mass and height and was used only to describe the sample.

Baseline characteristics were summarized as mean ± standard deviation, median [25th, 75th percentile], or frequency and percentage, as appropriate. Standardized mean differences were used to describe baseline differences between groups. Within-group changes from baseline to week 8 were examined using the Wilcoxon signed-rank test and were interpreted descriptively.

The between-group effect for each outcome was estimated as the baseline-adjusted mean difference in week-8 scores between PVT + CPT and CPT. Separate analysis of covariance models were fitted for the ODI, PSQI, and VAS, with treatment group and the corresponding baseline score as predictors [29]. Differences were calculated as PVT + CPT minus CPT; positive values favored CPT, and negative values favored PVT + CPT. Treatment-by-baseline interactions were examined to assess the homogeneity-of-regression-slopes assumption. Model residuals were examined for non-normality and heteroscedasticity. HC3 heteroscedasticity-consistent standard errors and corresponding 95% confidence intervals were used for the primary inference.

The ODI was designated as the primary outcome and evaluated at a two-sided alpha level of 0.05. PSQI and VAS were secondary outcomes. No adjustment for multiple comparisons was applied to the secondary outcomes, which were interpreted as exploratory. Stratified participant-level bootstrap confidence intervals based on 10,000 resamples and treatment-label permutation tests based on 20,000 permutations were used as sensitivity analyses.

No dated statistical analysis plan was established before data collection. The statistical approach was specified after data collection; HC3 standard errors were selected following residual inspection, and the bootstrap and permutation analyses were added after the principal models had been fitted. All 50 randomized participants had complete baseline and week-8 outcome data and were analyzed in their assigned groups. No outcome values were imputed.

## 3. Results

### 3.1. Participant Flow and Baseline Characteristics

**Figure 1:**
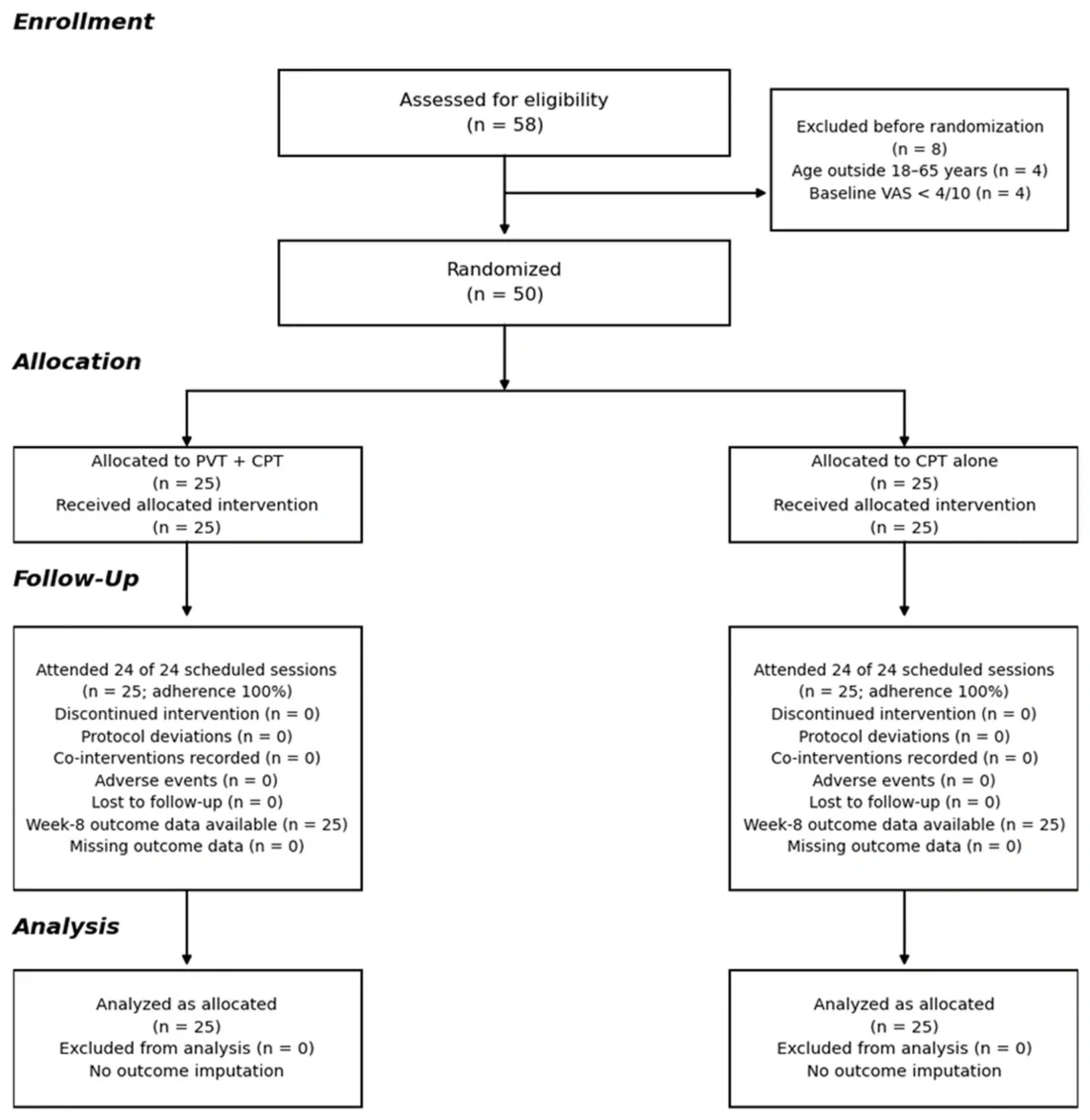
CONSORT flow diagram of participant recruitment, allocation, intervention adherence, discontinuation, follow-up, and analysis.

Of 58 individuals screened, 50 underwent randomization, with 25 assigned to each treatment group. All randomized participants completed the week-8 assessment and were analyzed in their assigned groups; no outcome values were missing or imputed (Figure 1).

Baseline characteristics are presented in Table 1. Thirty participants (60%) were women. Standardized mean differences were −0.28 for age, −0.16 for sex, +0.01 for body mass, +0.32 for height, −0.23 for body mass index, +0.04 for ODI, +0.08 for PSQI, and +0.35 for VAS. Each outcome model was adjusted for the corresponding baseline score.

All 25 participants in each group attended the 24 scheduled sessions. No participant discontinued treatment, and no protocol deviation was recorded. No adverse or unintended effect was observed by the treating physiotherapist or reported by participants.

Of the 58 individuals assessed for eligibility, eight were excluded before randomization: four did not meet the prespecified age criterion of 18–65 years, and four had baseline VAS pain intensity < 4/10. The remaining 50 participants were randomized equally to PVT + CPT or CPT alone. All randomized participants completed the week-8 assessment and were analyzed in their assigned groups. CPT, conventional physiotherapy; PVT, percussive vibration therapy; VAS, visual analogue scale. Treatment adherence is reported separately for each group. In the PVT + CPT group all 25 participants attended all 24 scheduled sessions (adherence 100%), and in the CPT group all 25 participants likewise attended all 24 scheduled sessions (adherence 100%). No participant in either group discontinued treatment and no protocol deviation was recorded in either group. No adverse or unintended effect was observed by the treating physiotherapist or reported by any participant in either group.

### 3.2. Primary Outcome

Table 2 presents baseline, week-8, and change scores. ODI values declined substantially in both groups, from a median of 62.0 to 20.0 in the PVT + CPT group and from 62.0 to 16.0 in the CPT group, and VAS values declined from 5.4 to 1.3 and from 5.2 to 2.3, respectively. Improvements of this magnitude were observed in both arms and, in the absence of an untreated comparator, cannot be attributed to either treatment program; they are reported descriptively and do not quantify the incremental treatment effect of PVT.

In the primary baseline-adjusted analysis, the week-8 ODI score was 2.59 points higher in the PVT + CPT group than in the CPT group (adjusted mean difference, +2.59; HC3 95% CI, +0.47 to +4.71; *p* = 0.017; Table 3). Because higher ODI scores indicate greater disability, the direction of the adjusted estimate favored CPT. Model-based adjusted week-8 means, estimated at the mean baseline score, were 21.17 (95% CI, 19.95 to 22.40) for PVT + CPT versus 18.59 (16.88 to 20.29) for CPT on the ODI, 4.81 (4.39 to 5.22) versus 4.55 (4.05 to 5.06) on the PSQI, and 1.23 (1.04 to 1.43) versus 2.39 (2.16 to 2.61) on the VAS.

The sensitivity analyses produced estimates in the same direction. The stratified bootstrap 95% confidence interval ranged from +0.74 to +4.52, and the treatment-label permutation test yielded *p* = 0.012. The adjusted difference was statistically significant but remained substantially below the conventional 10-point ODI interpretive benchmark. Because this benchmark primarily reflects within-person change, it was used only to contextualize the magnitude of the adjusted between-group estimate.

### 3.3. Secondary Outcomes

For sleep quality, the adjusted week-8 PSQI score was 0.25 points higher in the PVT + CPT group than in the CPT group (adjusted mean difference, +0.25; HC3 95% CI, −0.40 to +0.90; *p* = 0.451). The bootstrap 95% confidence interval ranged from −0.37 to +0.85, and the permutation *p*-value was 0.445. The confidence interval included zero and estimates favoring either group.

For pain intensity, the adjusted week-8 VAS score was 1.15 points lower in the PVT + CPT group than in the CPT group (adjusted mean difference, −1.15; HC3 95% CI, −1.45 to −0.85; *p* < 0.001). The bootstrap 95% confidence interval ranged from −1.44 to −0.86, and the permutation *p*-value was <0.001. The direction of the adjusted estimate favored PVT + CPT. However, VAS was a secondary outcome, and the adjusted difference was below the approximate 1.5-point within-person interpretive benchmark. Because VAS was a secondary outcome and no adjustment for multiple comparisons was applied, this finding was interpreted as exploratory.

Treatment-by-baseline interactions were not statistically significant for ODI (*p* = 0.182), PSQI (*p* = 0.380), or VAS (*p* = 0.213), providing no evidence of departure from the homogeneity-of-regression-slopes assumption.

## 4. Discussion

This trial assessed the added value of PVT within an eight-week multimodal physiotherapy program for CNLBP. The primary ODI analysis yielded a 2.59-point difference in favor of CPT, but both the estimate and its confidence interval were far below the conventional 10-point interpretive benchmark [25,26]. PSQI scores were similar between groups. Pain favored PVT + CPT by 1.15 points, although this secondary effect did not reach the approximate 1.5-point within-person benchmark [25]. The findings therefore showed a modest between-group difference in pain without additional benefits for disability or sleep quality.

The disability finding contrasts with Gorguluer and Menek, who reported greater ODI improvement after adding percussive vibration therapy to conventional therapy in individuals with non-specific low back pain [12]. Yang et al. also observed a small disability benefit when percussive vibration therapy was combined with core-stability exercise in firefighters with CNLBP, although disability was assessed with the Roland–Morris questionnaire and the between-group difference was modest [11]. The present study differed in its outpatient population, eight-week treatment duration, multimodal comparator, and baseline-adjusted analysis. More importantly, both groups received a substantial active physiotherapy program, thereby reducing the scope for an adjunctive modality to produce further functional improvement. The adjusted estimate provided no evidence of a clinically important incremental benefit of PVT for disability.

Recent trials of other massage-based adjuncts illustrate the importance of comparator selection. In a three-arm trial, connective-tissue and classical massage were compared with standard physiotherapy in a small chronic low back pain sample, with between-group effects varying across mobility, disability, and quality-of-life outcomes [30]. More directly, a 2025 placebo-controlled trial compared classical massage with placebo massage when both were added to conventional physiotherapy. Both groups improved in pain and disability, while the additional benefits attributable to the specific massage intervention were limited to selected outcomes [31]. These findings are consistent with the present active-comparator result: improvements observed during a multimodal treatment period do not necessarily demonstrate that the added manual or device-based component provides clinically important incremental functional benefit.

The secondary pain finding was directionally consistent with the PVT trials by Yang et al. and Gorguluer and Menek [11,12]. However, the adjusted difference in the present study was smaller than the selected interpretive benchmark and was not accompanied by improved disability. This distinction is clinically relevant because changes in pain intensity do not invariably translate into changes in activity limitation. Recent trials of massage-based interventions in CNLBP have similarly reported outcome-specific responses, with pain, mobility, disability, and quality-of-life measures not always changing in parallel [30,31]. The present findings therefore indicate a modest short-term between-group difference in pain without an additional disability benefit.

The two outcomes should not be treated as equivalent in this respect. The adjusted disability estimate lies far from its interpretive reference value, the upper confidence limit of +4.71 points remaining well inside the 10-point ODI reference, whereas the adjusted pain estimate of −1.15 points lies close to the 1.5-point pain reference, its lower confidence limit of −1.45 falling within 0.05 points of it. Describing both simply as falling below their reference values would obscure this asymmetry. The pain result nevertheless remains a secondary and exploratory finding: no adjustment was made for multiple comparisons, no sham or time-matched comparator was used, and the additional therapist contact in the experimental group cannot be separated from any specific effect of the device.

The absence of a PSQI difference is compatible with the available sleep literature. A meta-analysis of 19 randomized trials found that non-pharmacological interventions produced a small average improvement in sleep among adults with chronic low back pain, but the evidence was heterogeneous, of low certainty, and derived from interventions primarily designed to reduce pain rather than directly treat sleep disturbance [32]. More recent prognostic evidence indicates that sleep and low back pain are related over time, although certainty remains low and associations vary according to outcome and follow-up duration [33]. Against this background, a local percussive intervention without a sleep-specific component would not necessarily be expected to produce a measurable additional change in global sleep quality over eight weeks.

The active-comparator design determines the scope of inference. This trial did not compare PVT with no treatment and therefore does not estimate the absolute efficacy of the complete physiotherapy programs; it tested whether approximately 10 min of PVT added value beyond an established multimodal intervention. The clinically relevant conclusion is therefore limited to the incremental effect of adding PVT.

The specific contribution of the device cannot be separated from contextual components of treatment. Participants and treating physiotherapists were not blinded, no sham device was used, and the experimental group received approximately 10 min of additional therapist contact in every session, amounting to roughly four additional hours of supervised contact over the eight-week program. A systematic review of 64 randomized trials found small contextual effects of placebo conservative interventions on pain and physical function in musculoskeletal conditions, although the certainty of evidence was very low [34]. The pain difference observed here is of a magnitude compatible with such contextual effects, and the present design cannot separate the specific effect of the device from the effect of the additional contact time. A time-matched sham-device comparator would be required to do so.

### Strengths and Limitations

The study used a randomized active-comparator design, blinded outcome assessment, complete week-8 outcome data, baseline-adjusted ANCOVA, HC3 robust inference, and bootstrap and permutation sensitivity analyses. The consistency of the principal and sensitivity analyses supports the robustness of the statistical inference. The comparator also reflected a multimodal outpatient physiotherapy program, improving the clinical relevance of the incremental treatment question.

Several limitations should be considered. Participants and treating physiotherapists could not be blinded, the groups were not matched for treatment time or therapist attention, and no sham PVT procedure was included. Outcomes were self-reported, and follow-up ended at week 8; persistence of the pain difference is therefore unknown. The single-center setting and relatively small sample restrict external validity and precision for smaller effects. The sample-size calculation was based on a relatively large expected ODI effect, so the study was not designed to detect small functional differences. Adherence was documented from appointment records and was complete in both groups; complete adherence in a supervised eight-week program is unusual and reflects a single-center sample treated at one clinic, which may further limit generalizability. Treatments received outside the study clinic were not systematically recorded, the pressure applied during PVT was not quantified, and treatment fidelity was not formally monitored. Duration of low back pain, analgesic use, previous physiotherapy, occupational status and comorbidities were not recorded, which limits the characterization of the sample. Finally, the ODI and VAS benchmarks used for interpretation were developed primarily for within-person change and should not be regarded as thresholds for adjusted between-group effects.

## 5. Conclusions

In adults with CNLBP, adding PVT to an eight-week CPT program did not improve disability or sleep quality. The adjusted between-group difference in disability was small, favored CPT alone and was statistically significant; because no validated between-group minimal important difference exists for the ODI, this estimate is reported without a claim as to its clinical importance. A between-group difference in pain favored PVT + CPT, but the experimental group received approximately four additional hours of therapist contact over the program; the clinical relevance of this difference therefore remains uncertain. The pain finding should be confirmed in time-matched, sham-controlled trials with longer follow-up.

## Figures and Tables

**Table 1:** Baseline demographic and clinical characteristics of the randomized participants.

Characteristic	PVT + CPT (*n* = 25)	CPT (*n* = 25)	SMD
Age, years	36.5 ± 11.7	39.9 ± 12.6	−0.28
Female sex, *n* (%)	14 (56)	16 (64)	−0.16
Body mass, kg	71.0 ± 13.3	70.8 ± 13.5	+0.01
Height, cm	167.4 ± 8.8	164.6 ± 8.8	+0.32
Body mass index, kg/m^2^	25.2 ± 3.5	26.3 ± 5.5	−0.23
ODI, 0–100	62.0 [56.0, 70.0]	62.0 [50.0, 74.0]	+0.04
PSQI, 0–21	10.0 [9.0, 11.0]	10.0 [8.0, 12.0]	+0.08
VAS, 0–10	5.4 [5.2, 6.2]	5.2 [5.0, 5.4]	+0.35

Data are mean ± standard deviation, median [25th, 75th percentile], or *n* (%). SMDs compare PVT + CPT with CPT; continuous-variable SMDs were calculated from group means and pooled standard deviations, including for variables displayed as medians. BMI, body mass index; CPT, conventional physiotherapy; ODI, Oswestry Disability Index; PSQI, Pittsburgh Sleep Quality Index; PVT, percussive vibration therapy; SMD, standardized mean difference; and VAS, visual analogue scale.

**Table 2:** Descriptive outcome values at baseline and week 8.

Outcome	Group	Baseline	Week 8	Change
ODI, 0–100	PVT + CPT	62.0 [56.0, 70.0]	20.0 [18.0, 24.0]	−44.0 [−46.0, −38.0]
	CPT	62.0 [50.0, 74.0]	16.0 [14.0, 24.0]	−46.0 [−50.0, −34.0]
PSQI, 0–21	PVT + CPT	10.0 [9.0, 11.0]	5.0 [4.0, 5.0]	−5.0 [−7.0, −4.0]
	CPT	10.0 [8.0, 12.0]	4.0 [4.0, 5.0]	−6.0 [−8.0, −4.0]
VAS, 0–10	PVT + CPT	5.4 [5.2, 6.2]	1.3 [0.9, 2.0]	−4.2 [−4.5, −4.0]
	CPT	5.2 [5.0, 5.4]	2.3 [2.0, 2.8]	−3.0 [−3.3, −2.4]

Data are presented as median [25th, 75th percentile]. Change was calculated for each participant as the week-8 score minus the baseline score; the value reported is the median of these individual change scores and is not the difference between the baseline and week-8 group medians. Negative values indicate improvement. CPT, conventional physiotherapy; ODI, Oswestry Disability Index; PSQI, Pittsburgh Sleep Quality Index; PVT, percussive vibration therapy; and VAS, visual analogue scale.

**Table 3:** Baseline-adjusted between-group differences at week 8.

Outcome	Adjusted Difference (HC3 95% CI)	Bootstrap 95% CI	*p*-Value	Permutation *p*-Value
ODI, 0–100—primary outcome	+2.59 (+0.47 to +4.71)	+0.74 to +4.52	0.017	0.012
PSQI, 0–21—secondary outcome	+0.25 (−0.40 to +0.90)	−0.37 to +0.85	0.451	0.445
VAS, 0–10—secondary outcome	−1.15 (−1.45 to −0.85)	−1.44 to −0.86	<0.001	<0.001

Adjusted differences were calculated as PVT + CPT minus CPT, with adjustment for the corresponding baseline score. Positive values favor CPT and negative values favor PVT + CPT. Estimates are model-based adjusted mean differences with HC3 robust 95% confidence intervals. CI, confidence interval; CPT, conventional physiotherapy; HC3, heteroscedasticity-consistent covariance estimator; ODI, Oswestry Disability Index; PSQI, Pittsburgh Sleep Quality Index; PVT, percussive vibration therapy; VAS, visual analogue scale.

## Data Availability

The anonymized participant-level dataset and the analysis code used to produce all reported results are available from the corresponding author upon reasonable request and will be provided to the editorial office on request for the purpose of independent verification. The data are not publicly available because they contain information that could compromise participant privacy.

## Abbreviations

ANCOVA	Analysis of covariance
BMI	Body mass index
CI	Confidence interval
CNLBP	Chronic non-specific low back pain
CONSORT	Consolidated Standards of Reporting Trials
CPT	Conventional physiotherapy
HC3	Heteroscedasticity-consistent covariance estimator
ODI	Oswestry Disability Index
PSQI	Pittsburgh Sleep Quality Index
PVT	Percussive vibration therapy
RCT	Randomized controlled trial
SMD	Standardized mean difference
TENS	Transcutaneous electrical nerve stimulation
VAS	Visual analogue scale
